# Complementary feeding practices and associated factors among Mongolian children 6–23 months of age

**DOI:** 10.1111/mcn.12838

**Published:** 2020-08-24

**Authors:** Amynah Janmohamed, Munkhjargal Luvsanjamba, Bolormaa Norov, Enkhtungalag Batsaikhan, Batjargal Jamiyan, Jessica L. Blankenship

**Affiliations:** ^1^ UNICEF East Asia and Pacific Regional Office Toronto Canada; ^2^ UNICEF Mongolia Ulaanbaatar Mongolia; ^3^ Mongolia National Center for Public Health Ulaanbaatar Mongolia; ^4^ UNICEF East Asia and Pacific Regional Office Bangkok Thailand

**Keywords:** child, complementary feeding, malnutrition, Mongolia

## Abstract

Little is known about factors influencing children's dietary intake in Mongolia, a country undergoing rapid nutrition transition. Using nationally representative data from the 2017 Mongolia National Nutrition Survey, we assessed the nutritional status of children aged <2 years and examined household, maternal, and child factors associated with feeding practices among children aged 6–23 months (*n* = 938). Multivariable logistic regression models were used to identify predictors of minimum meal frequency (MMF), minimum dietary diversity (MDD), and minimum acceptable diet (MAD). The prevalence of child stunting (length/height‐for‐age *Z*‐score < −2 *SD*) was 6.3%, and the prevalence of overweight (weight‐for‐height *Z*‐score > +2 SD) was 16.8%. The prevalence of anaemia and iron deficiency was 39.0% and 32.2%, respectively, and 73.5% and 85.5% of children had inadequate vitamin A and vitamin D status, respectively. Of children aged 6‐23 months, 92.1% (*n* = 864) had MMF, 49.6% (*n* = 465) had MDD, and 43.8% (*n* = 411) achieved MAD. Increased household wealth was positively associated with all three indicators, whereas severe food insecurity was not associated with MMF, MDD, or MAD. Older child age (odds ratio, 95% CI: 1.09 [1.06, 1.12]; *p* < .001) and maternal dietary diversity (odds ratio, 95% CI: 2.36 [1.67, 3.34]; *p* < .001) were positively associated with child MDD. Nutrition‐specific and nutrition‐sensitive efforts are needed to improve the dietary quality of infants and young children in Mongolia and reduce the high burdens of child micronutrient deficiency and overweight in the country.

**Key messages**

- A high prevalence of micronutrient malnutrition and overweight exists among infants and young children across all socio‐demographic groups in Mongolia.
- Complementary feeding practices are suboptimal in Mongolia, with less than half of children receiving a minimum acceptable diet.
- Infants 6–11 months are at higher risk for poor‐quality diets compared with children 12–23 months of age.
- Household food security is not a major determinant of children's nutritional status in Mongolia.

## 1 INTRODUCTION

Food insecurity imperils the nutrition, health, and dignity of close to one billion people globally (FAO, IFAD, UNICEF, WFP, & WHO, 2017). In low‐income countries, food insecurity has primarily been associated with undernutrition, particularly among young children and women (FAO, IFAD, UNICEF, WFP, & WHO, 2017). However, increasing evidence suggests that food insecurity is also associated with overweight and obesity in low‐ and middle‐income countries (LMICs; Farrell, Thow, Abimbola, Faruqui, & Negin, 2017; Ford, Patel, & Venkat Narayan, 2017; Tzioumis & Adair, 2014). As food insecure households are generally thought to have insufficient food access, excess dietary energy intake of individuals living in such households is considered a nutritional paradox. An emerging double burden of malnutrition, characterized by caloric sufficiency, but underconsumption of nutrient‐rich foods, is increasingly recognized at the global, national, subnational, household, and individual levels (WHO, 2017). Recent evidence indicates a growing prevalence of child stunting and adult overweight coexisting within the same households, as well as the cooccurrence of micronutrient malnutrition and overweight at the individual level (Dang & Meenakshi, 2017; Gubert, Spaniol, Segall‐Corrêa, & Pérez‐Escamilla, 2017).

In Mongolia, rapid socio‐economic development in recent years has led to extensive migration from rural to urban areas, with ~70% of the population currently residing in urban settings and the majority living in the capital Ulaanbaatar (Mongolia Ministry of Health, 2017). This has created widespread urban poverty, with close to half of the Ulaanbaatar population now living in unplanned traditional tented housing (Ger) communities (Mongolia Ministry of Health, 2017). In rural areas, traditional pastoralism is the main source of livelihood, with the country's harsh climate substantially limiting agricultural productivity. These economic and environmental conditions represent major challenges to the food and nutrition security of the Mongolian population.

The country's changing demographic landscape has also led to altered food consumption patterns, with a shift away from traditional diets towards a Western‐influenced high‐energy and nutrient‐poor diet, exacerbated by the increased availability and affordability of low‐quality processed foods high in sugar, fat, and salt (Mongolia Ministry of Health, 2017). This has resulted in a marked increase in excess weight gain in all population groups, with an estimated 12% of children under 5 years and 30% of women 15–49 years overweight (Mongolia Ministry of Health, 2017). Nutritionally inadequate diets are also sustaining a high burden of micronutrient deficiencies in the population, particularly iron deficiency and anaemia among young children and women of reproductive age, and a high prevalence of vitamin D deficiency across all demographic groups (Mongolia Ministry of Health, 2017).

Dietary diversity is an indicator of dietary quality and the extent to which an individual's nutritional needs are being met. In Mongolia, little is known about factors influencing children's dietary intake and the extent to which household food insecurity affects infant and young child feeding practices, both of which have important policy‐relevant implications. The objective of this study was to examine complementary feeding practices and associated factors in a nationally representative sample of Mongolian children 6–23 months of age.

## 2 METHODS

### 2.1 Data collection

We utilized data from the 2017 Mongolia National Nutrition Survey conducted in the country's 21 provinces and four regions (Central, Eastern, Khangai, and Western) and the capital city Ulaanbaatar. The objective of the survey was to assess the nutritional status of the population by estimating the prevalence of nutrition‐ and diet‐related conditions in children aged 0–59 months, school‐aged children 6–11 years, pregnant and nonpregnant women aged 15–49 years, and men aged 15–49 years. A complete description of the survey design and methodology is published elsewhere (Mongolia Ministry of Health, 2017). Briefly, a two‐stage cluster *sampling design was used in which 30* clusters (communities) were randomly selected in each of the four regions and the capital city using probability proportional to size sampling. In each cluster, 15 households were randomly selected, for a total sample of 2,250 households. Household eligibility was based on a child 0–59 months of age living in the household.

Data collection occurred in all areas during September to November 2016. For children aged 0–59 months, weight (kg), height/length (cm), mid‐upper arm circumference (cm), haemoglobin (Hb) concentration (g/l), serum ferritin (μg/l), serum retinol (μmol/l), and serum 25‐hydroxyvitamin D (25[OH]D; ng/ml) were measured, adjusting for elevated C‐reactive protein and α1‐acid‐glycoprotein inflammation biomarkers. Hb concentration (g/l) was determined from a capillary (*finger‐tip*) *sample* using the HemoCue® Hb 201 photometer (Angelholm, Sweden). Current WHO guidelines were used to estimate the prevalence of anaemia (WHO, 2011a), iron deficiency (WHO, 2001), and vitamin A deficiency (WHO, 2011b). Estimates for vitamin D deficiency were based on Holick et al. clinical practice guidelines (Holick, 2011). Children's weight and height status was assessed based on the WHO Child Growth Standards (WHO & UNICEF, 2009). For mothers, weight (kg) and height (cm) were measured and anthropometric status was assessed based on the WHO body mass index (BMI) classification for underweight, normal weight, overweight, and obese (WHO, 2018).

Household food security status was determined using the Household Food Insecurity Access Scale (Coates, Swindale, & Bilinsky, 2007), which includes nine dichotomous (yes/no) questions examining the occurrence of specific events according to an increasing level of severity, and corresponding frequency‐of‐occurrence questions to identify how often the event took place during the previous 30 days. The sum of responses is used to calculate a continuous Household Food Insecurity Access Scale score, from which a categorical assessment of food insecurity (mild, moderate, or severe) is established for each household. The dietary intake of children 6–23 months and their mothers was assessed from 24‐hr dietary recall data collected during the survey. Children's dietary adequacy was evaluated according to the WHO indicators of minimum meal frequency (MMF), minimum dietary diversity (MDD), and minimum acceptable diet (MAD) for assessing the appropriateness of infant and young child feeding practices (WHO, 2008). The MDD indicator for women (FAO & FHI 360, 2016) was used to assess maternal dietary quality.

### 2.2 Data analysis

Bivariate analyses were conducted to identify factors associated with *children's nutritional status*. We created multivariable logistic regression models to examine predictors of MMF, MDD, and MAD for children 6–23 months of age. Model covariates were selected based on a significant (*p* < .05) bivariate relationship and/or were factors known to be associated with children's dietary intake and/or nutritional status. The models were adjusted for the cluster sampling design. Results are expressed as odds ratios (OR; 95% CI), and a two‐sided significance level was used (*α* = .05). Analyses were conducted using IBM SPSS Version 23.0 (IBM Corporation, Armonk, NY).

## 3 RESULTS

A total of 938 children 6–23 months were included in the analysis. Two‐thirds of households (68.4%; *n* = 642) were located in urban areas and 31.6% (*n* = 296) in rural areas (Table 1). The majority of households (60.1%; *n* = 563) were in the three lowest wealth quintiles, with 22.6% (*n* = 211) in the poorest wealth category. Overall, 63.4% (*n* = 595) of households had some level of food insecurity, with 15.5% (*n* = 146) mildly food insecure and 27.4% (*n* = 257) and 20.5% (*n* = 192) experiencing moderate and severe food insecurity, respectively.

**Table 1:** Household, child, and maternal characteristics

	*n*	%
*Household*		
Region		
Central	144	15.4
Eastern	53	5.7
Khangai	164	17.4
Western	107	11.4
Ulaanbaatar	470	50.1
Area		
Rural	296	31.6
Urban	642	68.4
Food security status^a^		
Food secure	343	36.6
Mild food insecurity	146	15.5
Moderate food insecurity	257	27.4
Severe food insecurity	192	20.5
Wealth quintile^b^		
Poorest	185	19.7
Second	190	20.2
Third	164	17.5
Fourth	188	20.0
Wealthiest	211	22.6
Improved drinking water^b^	875	93.3
Improved sanitation facility^b^	629	67.1
*Child*		
Age group		
6–11 months	337	35.9
12–23 months	601	64.1
Sex		
Female	478	50.9
Male	460	49.1
Anthropometric status		
Stunted^c^	58	6.3
Wasted^d^	13	1.4
Underweight^e^	4	0.4
Overweight^f^	155	16.8
Micronutrient status		
Anaemia^g^	358	39.0
Iron deficiency^h^	231	32.2
Vitamin A deficiency^i^	109	15.1
Vitamin D deficiency^j^	448	58.6
*Maternal*		
Age group		
15–19 years	11	1.3
20–29 years	397	47.9
30–39 years	358	43.3
40–49 years	62	7.5
Education completed		
None	4	0.5
Primary	15	1.9
Secondary	366	44.1
Higher	443	53.5
Anthropometric status		
Height < 150 cm	64	7.7
Underweight^k^	35	4.2
Normal weight	448	54.2
Overweight^l^	229	27.7
Obese^m^	114	13.9
Anaemia^n^	66	15.9

a Measured by Household Food Insecurity Access Scale (Coates et al., 2007).

b Based on methodology used in the 2013 Mongolia Social Indicators Survey (National Statistical Office of Mongolia, 2013).

c Length/height‐for‐age < −2 *SD* below WHO Child Growth Standards median.

d Weight‐for‐height < −2 *SD* below WHO Child Growth Standards median.

e Weight‐for‐age < −2 *SD* below WHO Child Growth Standards median.

f Weight‐for‐length/height > +2 *SD* above WHO Child Growth Standards median.

g Altitude‐adjusted Hb < 110 g/l.

h Serum ferritin < 12 μg/l.

i Serum retinol ≤ 0.70 μmol/l.

j Serum 25(OH)D < 20 ng/ml.

k BMI < 18.5 kg/m^2^.

l BMI ≥ 25 kg/m^2^.

m BMI ≥ 30 kg/m^2^.

n Hb < 120 g/l adjusted for altitude and smoking.

Among children 6–23 months of age, 35.9% (*n* = 337) were aged 6–11 months and 64.1% (*n* = 601) were aged 12–23 months (Table 1). Boys (49.1%; *n* = 460) and girls (50.9%; *n* = 478) were equally represented. The prevalence of child underweight (weight‐for‐age WAZ < −2 SD) was 0.4% (*n* = 4), the prevalence of child wasting (weight‐for‐height *Z*‐score < −2 SD) was 1.4% (*n* = 13), and the prevalence of child stunting (height‐for‐age *Z*‐score < −2 SD) was 6.3% (*n* = 58). The prevalence of child overweight (weight‐for‐height *Z*‐score > +2 SD) was 16.8% (*n* = 155).

More than one‐third (39.0%; *n* = 358) of children aged 6–23 months were anaemic (Hb < 110 g/l); 55.3% (*n* = 198) of the anaemic children had mild anaemia (Hb 100–109 g/l), 41.6% (*n* = 149) had moderate anaemia (Hb 70–99 g/l), and 3.1% (*n* = 11) had severe anaemia (Hb < 70 g/l). Regarding micronutrient status, 32.2% (*n* = 231) were iron‐deficient (serum ferritin <12 μg/l), 73.5% (*n* = 529) were vitamin A‐deficient/insufficient (serum retinol ≤1.05 μmol/l), and 85.5% (*n* = 653) had vitamin D deficiency/insufficiency (serum 25[OH]D < 30 ng/ml). Low vitamin D status was prevalent in all regions, wealth quintiles, and child age groups. Among mothers, 91.2% (*n* = 755) were 20–39 years of age and 97.6% (*n* = 809) had completed secondary or higher education (Table 1). Approximately 30% of all mothers (27.7%; *n* = 229) were overweight (BMI ≥ 25.0 kg/m^2^), 13.9% (*n* = 114) were obese (BMI ≥ 30.0 kg/m^2^), and 15.9% (*n* = 66) were anaemic (Hb < 120 g/l).

When analysing the nutritional status of children as a function of household food security, the percentages of stunted children, anaemic children, and children having iron and vitamin D deficiency were similar across food security categories (Table 2). However, the proportion of overweight children was significantly higher in food secure/mildly food insecure households, as compared with moderately and severely food insecure households (*p* < .001), and the prevalence of child vitamin A deficiency was highest in severely food insecure households (*p* = .033).

**Table 2:** Child nutritional status according to household food security status

	Stunted^a^ *n* (%)	Overweight^b^ *n* (%)	Anaemic^c^ *n* (%)	Iron deficient^d^ *n* (%)	Vitamin A deficient^e^ *n* (%)	Vitamin D deficient^f^ *n* (%)
Food secure/mildly food insecure households^g^	27 (5.6)	114 (23.8) *	180 (37.7)	112 (30.9)	55 (15.2)	228 (58.8)
Moderately food insecure households^g^	15 (5.8)	23 (9.1)	103 (40.9)	64 (30.6)	23 (11.0)	123 (56.7)
Severely food insecure households^g^	16 (8.5)	18 (9.5)	75 (39.7)	55 (37.4)	31 (21.1)*	97 (61.0)

a Length/height‐for‐age < −2 *SD* below WHO Child Growth Standards median.

b Weight‐for‐length/height > +2 *SD* above WHO Child Growth Standards median.

c Altitude‐adjusted Hb < 110 g/l.

d Serum ferritin < 12 μg/l.

e Serum retinol ≤ 0.70 μmol/l.

f Serum 25(OH)D < 20 ng/ml.

g Measured by Household Food Insecurity Access Scale (Coates et al., 2007).

* *p* < .05 (chi‐squared test).

The 24‐hr dietary recall revealed nearly all children aged 6–23 months (92.1%; *n* = 864) received MMF (90.8% for breastfeeding and 95.7% for nonbreastfeeding children; *p* = .012), and only half (49.6%; *n* = 465) of children received MDD. The MAD assessment indicated that 43.8% (*n* = 411) of children were provided both appropriate meal frequency and dietary diversity the previous day. The percentage of children receiving MMF was similar across food security categories; however, significantly lower proportions of children had adequate dietary diversity (*p* = .031) and an overall acceptable diet (*p* < .001) in severely food insecure households (Table 3). The maternal 24‐hr dietary recall revealed that 70.1% (*n* = 1,348) of mothers had MDD the previous day, with a lower proportion having a minimally diverse diet in severely food insecure households, as compared with food secure/mildly food insecure and moderately food insecure households (*p* < .001; Table 3).

**Table 3:** Dietary indicators of children 6–23 months and mothers according to household food security status

	Dietary assessment indicator			
Household food security status^a^	Child minimum meal frequency^b^	Child minimum dietary diversity^c^	Child minimum acceptable diet^a^, ^d^	Maternal minimum dietary diversity*, e
Food secure/mild food insecurity	455 (93.0)	255 (52.1)	228 (46.5)	733 (77.2)
Moderate food insecurity	237 (92.6)	131 (51.0)	123 (47.9)	367 (69.4)
Severe food insecurity	171 (89.5)	79 (41.1)*	60 (31.2)*	248 (55.9)*

a Measured by Household Food Insecurity Access Scale (Coates et al., 2007).

b Proportion of children 6–23 months who received the appropriate number of meals/snacks/milk feeds in the previous 24 hr.

c Proportion of children 6–23 months who consumed food items from at least four of these seven food groups in the previous 24 hr: grains, roots, and tubers; legumes and nuts; dairy products; flesh foods (meat, fish, poultry, and liver/organ meats); eggs; vitamin‐A rich fruits and vegetables; and other fruits and vegetables.

d Proportion of children 6–23 months who received the appropriate number of meals/snacks/milk feeds and consumed food items from at least 4/7 of the above‐mentioned food groups in the previous 24 hr.

e Proportion of mothers who consumed food items from at least 5 out of the following 10 food groups in the previous 24 hr: grains, white roots, tubers, plantains; pulses (beans, peas, lentils); nuts and seeds; dairy; meat, poultry, fish; eggs; dark green leafy vegetables; other vitamin A‐rich fruits and vegetables; other vegetables; and other fruits.

* *p* < .05 (chi‐squared test).

Among anaemic children aged 6–23 months, 90.5% (*n* = 325) received MMF, 45.3% (*n* = 162) had MDD, and 41.2% (*n* = 148) had MAD. The prevalence of MMF, MDD, and MAD among nonanaemic children was 93.2% (*n* = 522), 52.3% (*n* = 293), and 45.2% (*n* = 253), respectively, with a significantly higher prevalence of MDD among nonanaemic children (*p* = .037). Among overweight children aged 6–23 months, 92.2% (*n* = 142) had MMF, 44.5% (*n* = 69) received MDD, and 40.9% (*n* = 63) had MAD, with 92.3% (*n* = 709), 50.4% (*n* = 387), and 44.0% (*n* = 338) of nonoverweight children attaining MMF, MDD, and MAD, respectively. In addition, among overweight children, 31.9% were anaemic, 26.6% were iron‐deficient, and 11.8% and 55.1% had vitamin A and vitamin D deficiency, respectively.

Children 6–23 months in wealthier households were more likely to receive MMF, and male children were less likely to receive the appropriate number of meals/snacks the previous day (OR, 95% CI: 0.53 [0.31, 0.89]; *p* = .017), as compared with female children (Table 4). Children in wealthier households were also more likely to receive MDD, as compared with children in the poorest households (Table 5). Child age (OR, 95% CI: 1.09 [1.06, 1.12; *p* < .001]) and maternal MDD (OR, 95% CI: 2.36 [1.67, 3.34]; *p* < .001) were also significant predictors of children's MDD in the adjusted model. For the composite indicator of MAD, children in the wealthiest households (OR, 95% CI: 1.95 [1.13, 3.36]; *p* = .016) and those whose mothers had MDD the previous day (OR, 95% CI: 2.43 [1.71, 3.45]; *p* < .001) were more likely to be provided the appropriate quantity and quality of foods during the preceding 24 hr (Table 6). Older child age was of marginal significance with respect to receiving an acceptable diet. Severe household food insecurity was not associated with meal frequency, diversity, or the overall acceptability of children's diets.

**Table 4:** Association between independent variables and minimum meal frequency among children 6–23 months^a^

Variables	OR	95% CI	*p* value
Rural household	1.27	0.65, 2.47	.487
First wealth quintile (Reference)	1.00		
Second wealth quintile	1.26	0.60, 2.66	.549
Third wealth quintile	1.09	0.51, 2.35	.826
Fourth wealth quintile	2.59	1.02, 6.56	.045*
Fifth wealth quintile	3.74	1.30, 10.78	.015*
Severe household food insecurity	0.84	0.46, 1.53	.561
Number of household members	1.22	0.96, 1.56	.103
Child age	1.03	0.98, 1.08	.238
Male child	0.53	0.31, 0.89	.017*
Maternal age	1.00	0.95, 1.05	.904
Maternal minimum dietary diversity	1.41	0.82, 2.44	.215

Abbreviations: CI, confidence interval; OR, odds ratio.

a Categorical reference variables: rural household, first wealth quintile (poorest households), no severe food insecurity, female child, and no maternal minimum dietary diversity. Continuous variables: number of household members, child age, and maternal age.

* *p* < .05.

**Table 5:** Association between independent variables and minimum dietary diversity among children 6–23 months^a^

Variables	OR	95% CI	*p* value
Rural household	1.09	0.73, 1.65	.670
First wealth quintile (Reference)	1.00		
Second wealth quintile	0.89	0.53, 1.49	.646
Third wealth quintile	1.72	1.03, 2.88	.039*
Fourth wealth quintile	2.23	1.32, 3.76	.003*
Fifth wealth quintile	2.49	1.43, 4.33	.001*
Severe household food insecurity	0.98	0.67, 1.44	.930
Number of household members	1.07	0.93, 1.24	.351
Child age	1.09	1.06, 1.12	<.001*
Male child	0.85	0.63, 1.15	.282
Maternal age	1.03	1.00, 1.05	.082
Maternal minimum dietary diversity	2.36	1.67, 3.34	<.001*

Abbreviations: CI, confidence interval; OR, odds ratio.

a Categorical reference variables: rural household, first wealth quintile (poorest households), no severe food insecurity, female child, and no maternal minimum dietary diversity. Continuous variables: number of household members, child age, and maternal age.

* *p* < .05.

**Table 6:** Association between independent variables and minimum acceptable diet among children 6–23 months^a^

Variables	OR	95% CI	*p* value
Rural household	1.11	0.74, 1.66	.618
First wealth quintile (Reference)	1.00		
Second wealth quintile	0.86	0.51, 1.44	.557
Third wealth quintile	1.37	0.82, 2.29	.233
Fourth wealth quintile	1.59	0.95, 2.68	.078
Fifth wealth quintile	1.95	1.13, 3.36	.016*
Severe household food insecurity	0.72	0.49, 1.05	.088
Number of household members	1.00	0.87, 1.15	.964
Child age	1.04	1.01, 1.07	.008*
Male child	0.80	0.60, 1.07	.137
Maternal age	1.02	0.99, 1.05	.202
Maternal minimum dietary diversity	2.43	1.71, 3.45	<.001*

Abbreviations: CI, confidence interval; OR, odds ratio.

a Categorical reference variables: rural household, first wealth quintile (poorest households), no severe food insecurity, female child, and no maternal minimum dietary diversity. Continuous variables: number of household members, child age, and maternal age.

* *p* < .05.

## 4 DISCUSSION

This study revealed a high prevalence of food insecurity among Mongolian households. One in five households experienced severe food deprivation, with a larger proportion of food insecure households in urban areas. The association between urbanization and low food security has been observed in low‐income countries (Dangura & Gebremedhin, 2017; Matuschke & Kohler, 2014; Ruel, Garrett, Yosef, & Olivier, 2017; Szabo, 2016). Although more than 70% of the poorest households experienced some level of food insecurity, approximately 40% of households in the wealthiest quintile also experienced moderate or severe food insecurity, suggesting that factors other than food availability and affordability are likely influencing children's diets in this context.

Our study findings indicate that recommended complementary feeding practices are not being achieved in Mongolia. The fact that almost all children had appropriate meal frequency, whereas only half received a minimally diverse diet (provided foods from ≥4 food groups), suggests that children's inability to achieve an adequate diet is primarily due to limited dietary diversity and low consumption of nutrient‐rich foods, rather than insufficient caloric intake. Children consuming foods from at least four food groups are more likely to consume at least one animal‐source food and one fruit or vegetable, in addition to a starchy staple food. As expected, a significantly lower proportion of children achieved adequate dietary diversity and an overall acceptable diet in severely food insecure households. A study conducted among children and adolescents in Mexico revealed that food insecurity was positively associated with intake of refined grains and inversely associated with consumption of fruits, vegetables, and protein foods (Rodríguez, Mundo‐Rosas, Méndez‐Gómez‐Humarán, Pérez‐Escamilla, & Shamah‐Levy, 2017).

However, as the prevalence of child stunting, anaemia, iron, and vitamin D deficiency was comparable across food secure and food insecure households, our findings suggest that children's vulnerability to dietary inadequacy exists across socioeconomic strata. The lack of effect of household size on children's feeding practices also points to factors other than food/resource availability that may be underlying nutritional inadequacy among Mongolian children. It would be useful to examine IYCF practices across a variety of food security experiences to understand influencers of food choices and feeding behaviours such as parental knowledge, cultural norms, and intrahousehold food allocation practices in the Mongolian context.

In our study, the likelihood of achieving adequate dietary diversity increased with child age, with infants aged 6–11 months at higher risk for a poor quality diet compared with children 12–23 months of age. The transition from exclusive breastfeeding to complementary feeding is challenging for many mothers and is commonly associated with delayed introduction of first foods and provision of inadequate quantity and/or quality of food for optimal child growth and development (Abeshu, Lelisa, & Geleta, 2016; Dewey, 2013; Issaka et al., 2015a (Issaka et al., 2015b); Mitchodigni et al., 2017). The fact that maternal dietary diversity was positively associated with children's dietary diversity suggests that mothers who consume items from a variety of food groups are more likely to ensure their children do so as well.

The low prevalence of child underweight, wasting, and stunting reflects the substantial improvements in child growth achieved by the government of Mongolia in recent years (Joshi, Bolorhon, Narula, Zhu, & Manaseki‐Hollan, 2017; Mongolia Ministry of Health, 2017). These are laudable successes and provide compelling evidence that a sizable reduction in child stunting is within reach in low‐income countries. However, Mongolia is now faced with a rapidly increasing burden of adult and child overweight and obesity. The ~10% overweight prevalence among children in moderately and severely food insecure households and the fact that less than half of overweight children had an acceptable diet in our study were unexpected. However, emerging data from LMICs indicate an association between food insecurity and consumption of energy‐dense foods, resulting in higher risks for overweight, obesity, and micronutrient malnutrition (Jomaa, Naja, Cheaib, & Hwalla, 2017; Schlüssel, Silva, Pérez‐Escamilla, & Kac, 2013; Vuong, Gallegos, & Ramsey, 2015). Low‐resource households tend to allocate a larger share of their income to food purchases, resulting in predominantly higher consumption of cereals/tubers and increasingly affordable highly processed poor quality foods at the expense of a diverse and nutritious diet (Shamah‐Levy et al., 2017; World Bank, 2018). The fact that overweight is being established at a young age in Mongolia has serious implications for the onset of diabetes, hypertension, and other chronic illnesses in early life (WHO & FAO, 2003).

Given that both undernutrition and overweight are rooted in dietary inadequacy, increasing the nutrient density of children's diets should be a key focus of Mongolia's nutrition policy agenda. This necessitates creating enabling community and household environments for nutrition‐promoting behaviours and implementing both nutrition‐specific and nutrition‐sensitive programmes to address the direct and underlying causes of malnutrition such as lack of access to safe water and health services. Key components should include providing mothers and caregivers with appropriate messages emphasizing the importance of a diversified diet for their children and promoting the consumption of locally available nutrient‐dense foods. In addition, to ensure young children meet their requirements for essential micronutrients, the revitalization and strengthening of national vitamin A and vitamin D supplementation programmes, as well as the provision of vitamins and minerals through in‐home fortification of complementary foods with micronutrient powders (e.g., Sprinkles), should be considered in Mongolia, as they offer simple and cost‐effective approaches for improving children's nutritional status (WHO, 2016).

Furthermore, strengthening the national food stamp programme through improved targeting of poor urban and rural households and increasing the quality of the eligible food basket is recommended as part of the country's social protection strategy to provide safety nets for households experiencing the greatest burden of food insecurity. Through enabling access to a greater variety of foods, food stamps have shown to be effective for reducing vulnerability to food insecurity and increasing dietary diversity in urban areas of Mongolia (Asian Development Bank, 2014). For maximum benefit, the food stamp programme should encompass the period from pregnancy up to 2 years. Finally, recently legislated national fortification measures for wheat flour and milk constitute enormous steps towards reducing micronutrient malnutrition in the Mongolian population.

In addition to these food‐based, supplementation, and fortification strategies, investing in the prevention of childhood overweight and obesity is important for reducing the risk of developing these conditions and comorbidities into adolescence and adulthood. This necessitates a broad approach addressing the socioeconomic and environmental influences governing food choices. Key to preventing excess child weight gain are parental messages promoting healthy and nutritious foods, encouraging good feeding habits, and discouraging the purchase of high‐fat and high‐sugar “junk” foods for their children. Mandatory *nutrition labelling* on prepackaged *foods* should be considered to help parents make healthier food choices. In addition, market‐based strategies such as taxes on sugar‐sweetened beverages and restricted advertising to children to reduce obesogenic influences, as well as family focused physical activity programmes, should be considered to prevent and reduce overweight in the Mongolian population (WHO, 2012).

Our study included a large nationally representative sample of children and mothers in Mongolia. As this was a cross‐sectional survey, we were not able to infer causation from observed associations between dependent and outcome‐influencing variables. Survey data were collected during a 2‐month period at the end of autumn/beginning of winter and, consequently, may not represent children's dietary patterns and risk of nutritional inadequacy during other seasons in Mongolia. Finally, as the dietary quality assessment was based on foods children consumed during a 24‐hr period, it may not reflect the usual feeding habits of children who were ill or experiencing other atypical events. However, as consistent associations were observed across the three diet‐related indicators measured, this unlikely affected our results.

Nutritionally inadequate diets, consisting of limited variety and higher consumption of energy‐dense nutrient‐poor foods, are contributing to the high dual burden of malnutrition among Mongolian children. Multisectoral equity‐focused efforts are necessary to improve children's dietary intake amidst shifting food consumption patterns associated with Mongolia's nutrition transition.

## CONFLICTS OF INTEREST

The authors declare that they have no conflicts of interest.

## SOURCE OF FUNDING

None declared.

## CONTRIBUTIONS

AJ and JLB designed the study; AJ and JLB conducted the data analysis; AJ, ML, NB, BE, JB, and JLB interpreted the data; AJ and JLB drafted the manuscript; and ML, NB, BE, and JB contributed to the preparation of the manuscript. All authors approved the final manuscript. AJ and JLB had primary responsibility for the final content. The manuscript's contents are solely the responsibility of the authors and do not necessarily represent the official views of UNICEF or the National Center for Public Health in Mongolia.
